# A Systematic Review of Interventions Addressing the Primary Prevention of Violence Against Women With Disability

**DOI:** 10.1177/15248380231175932

**Published:** 2023-06-05

**Authors:** Georgina Sutherland, Jen Hargrave, Lauren Krnjacki, Gwynnyth Llewellyn, Anne Kavanagh, Cathy Vaughan

**Affiliations:** 1 University of Melbourne, Australia; 2 University of Sydney, Australia

**Keywords:** vulnerability to abuse, developmentally delayed, domestic violence, intervention/treatment, domestic violence

## Abstract

Women with disability experience significantly more violence and abuse than their nondisabled peers. Efforts to implement, evaluate, and scale-up strategies to prevent violence against women are rapidly expanding, but we know less about “what works” to prevent violence against women with disability. While secondary and tertiary prevention aim to identify violence early and prevent further occurrence, this review focuses on primary prevention. In the disability services sector, primary prevention is sometimes referred to as safeguarding and covers a range of activities that aim to address the underlying determinants of violence to prevent it from happening in the first place. The aim of this review is to identify and synthesize research on evaluated interventions addressing the primary prevention of violence against women with disability and explore evidence about their quality and effectiveness. A systematic search across the bibliographic databases of Medline, CINAHL, Embase, and PsychInfo for peer-reviewed literature published in English on or after January 1, 2010, yielded 483 papers of potential interest. Twelve studies met the inclusion criteria and were considered for review. Data were extracted and the quality of the studies was assessed using the Quality Assessment Tool for Quantitative Studies. Most studies reported outcomes from pre- and post-test research designs and received a weak rating of quality. Although interventions targeting awareness, knowledge, and skill development showed evidence of effectiveness, there is a distinct lack of program development that draws on known risk factors for violence such as the intersection of ableism and gender inequality.

## Introduction

Violence against women is a pervasive global public health concern and a violation of human rights that has well-established and long-lasting physical and mental health and social impacts (World Health Organization, 2021). While violence affects people from all cultures, ages, and socioeconomic groups, the extent, nature, and dynamics of violence are not evenly distributed across the community. Not only is use of, and exposure to, violence heavily gendered—largely perpetrated by men against women—but there is also a range of additional factors that are known to elevate risk. An expanding evidence base indicates the extent and severity of violence are higher among women experiencing multiple forms of inequality and disadvantage (Brownridge, 2006; Nixon & Humphreys, 2010). This is now well documented for women with disability who experience violence and abuse at significantly higher rates than women without disability (Basile et al., 2016; Breiding & Armour, 2015; Chirwa et al., 2020; Mailhot Amborski et al., 2022; Plummer & Findley, 2012). For women with disability, gender- and disability-based violence intersects and compounds experiences of violence (Dowse et al., 2016). Not only are the rates of violence higher, but violence tends to be perpetrated in more diverse ways, manifesting in multiple, interrelated, and recurring forms (Frohmader et al., 2015).

Globally, the need to strengthen efforts to prevent violence against women is well recognized (Ellsberg et al., 2015; García-Moreno et al., 2015; Michau et al., 2015). In 2015, all governments of the United Nations committed to eliminating violence against women by 2030 (United Nations, 2015). Although ambitious in its target, the goal of elimination has accelerated efforts to identify the underlying determinants and contributing factors for violence perpetration and what works to prevent it from happening in the first place.

Informed by a public health framework, interventions to address violence are commonly divided into three main types: primary, secondary, and tertiary prevention (Our Watch, 2021). To date, most attention has been paid to secondary and tertiary prevention initiatives that attend to early identification and intervention, and work with victim/survivors in crisis response, support, and recovery from violence. Although primary prevention—stopping violence *before* it occurs—is much harder to implement and evaluate, it is now recognized as critical for achieving sustained reductions in violence (Ellsberg et al., 2015). Shifting the paradigm from service response and protection to prevention is also evident in policies and practices to safeguard people with disability from violence and abuse (Cortis & Van Toorn, 2022).

To date, evidence-based approaches to primary prevention have largely focused on gender inequality as the key social determinant or driver of violence against women (Jewkes et al., 2015; Krantz & Garcia-Moreno, 2005; Webster et al., 2021). This includes, for example, interventions that target gender norms and power imbalances, such as relationship dynamics; household, workplace, and community structures; access to financial, employment, and other social protections, as well as universal interventions that challenge harmful attitudes and stereotypes about women and men in schools, sports, popular culture, media, and advertising. Since Heise (1998) first published and advocated for a socio-ecological approach to advance action on gender-based violence, evidence has increasingly shown that interventions that target multilayers of influence at the individual, relationship, community, and societal levels are more effective than those that target single-level risk factors in isolation (Casey & Lindhorst, 2009; Christofides et al., 2020; Jewkes et al., 2021). Education programs delivered within school settings, for example, have been a particular focus of primary prevention efforts internationally, largely because of their potential to address the harmful behaviors and attitudes that lead to violence early in young people’s development in a setting that involves multilayers of influence including peers, teachers, parents/carers, and the wider school community.

While there has been less empirical investigation into the specific underlying factors that drive violence against women with disability, there is an emerging international body of evidence that points to where primary prevention strategies might be targeted. Conceptualized in the literature as gendered disability violence, the evidence demonstrates that ableism and sexism intersect to create the social conditions that enable and tolerate violence against women with disability (Healy et al., 2008; Meer & Combrinck, 2015; Platt et al., 2017; Woodlock et al., 2014). Research shows these social conditions are reenacted at the community, relationship, and individual levels resulting in a range of intersecting factors that place women with disability at particular risk of violence (Frohmader et al., 2015).

These intersecting factors may include, for example, gendered drivers of violence, such as rigid gender norms, stereotypes, and ableist drivers of violence such as social segregation and exclusion from participation in decision-making, community life, education, and employment.

With an evidence base about what drives violence against women with disability, and a clear imperative that these factors need to be addressed for primary prevention approaches to be effective, it is surprising that there remains a limited understanding about “what works.” Several prior reviews of the literature have explored violence prevention strategies for people with disability without a specific focus on primary prevention (Araten-Bergman & Bigby, 2020; Araten-Bergman et al., 2017; Mikton et al., 2014; Stobbe et al., 2021). The most comprehensive review to date is a systematic review undertaken by Mikton et al. (2014) who brought together evidence on the effectiveness of interventions in prevention and response, inclusive of all forms of interpersonal violence against all people with disability. In this review, 10 studies met the criteria for inclusion; all were rated as methodologically weak and none of the interventions were considered effective.

Other reviews of the literature on prevention have been narrower in scope than that of Mikton et al., either by selecting studies in which interventions target only some types of violence (e.g., sexual violence, intimate partner violence; Barger et al., 2009); address specific impairments (e.g., intellectual disability; Araten-Bergman & Bigby, 2020; Stobbe et al., 2021); or focus on certain settings only (e.g., residential/group homes; Araten-Bergman et al., 2017). Despite clear evidence about the disproportionately high rates of violence experienced by women with disability, to our knowledge there is only one prior review of the literature with a focus on women with disability and violence prevention (Barger et al., 2009). This review summarized the literature on initiatives to prevent sexual violence but it only included interventions designed *for* women with disability (e.g., building knowledge and skills to prevent violence). It specifically excluded studies that engaged with other “audiences” or targets for primary prevention programming. While understanding the effectiveness of prevention initiatives for women with disability is important, there is a notable gap in the evidence base about effective programming to mitigate the underlying drivers of violence against women with disability that sits across the broader population, community, relationship, and individual levels.

This review aims to address this knowledge gap by identifying, appraising, and providing evidence on evaluated interventions addressing the primary prevention of violence against women with disability. This review critically evaluates the included studies and explores evidence about the quality and effectiveness of the interventions they describe.

## Method

We conducted this review according to the PRISMA (Preferred Reporting Items for Systematic Reviews and Meta-Analyses) statement (Liberati et al., 2009). Data were extracted using COVIDENCE, a web-based screening and data extraction tool (www.covidence). Covidence is one of the tools recommended by Cochrane Collaboration for the management of systematic reviews. The search strategy, eligibility criteria, study selection, data extraction, and assessment of study quality/risk of bias are described below.

### Defining a Primary Prevention Intervention

Internationally, primary prevention of violence against women is a now central priority of policy development, with a concomitant increase in funding and programmatic activity. This focus on addressing casual factors for violence represents a real shift in how prevention work is conceived. In this burgeoning space, however, there has been a lack of clarity about what constitutes primary prevention, particularly in comparison to early intervention (where perpetrators are a key focus).

For the purpose of this review, we considered a “primary prevention intervention” as an intentionally implemented strategy or program that aims to stop violence before it starts by addressing the underlying causes, behaviors, and attitudes that lead to the perpetration of violence (Our Watch, 2021). Therefore interventions may be designed *for* women with disability to increase knowledge and skills to recognize violence, disrespect, or controlling behaviors, but equally interventions in primary prevention may include both women and men with disability, family, friends, support workers, service providers, or the wider community across various settings and sectors (e.g., interventions in workplaces to promote gender and disability-inclusive practices). Primary prevention initiatives may target the whole population (e.g., social marketing) or specific populations groups, such as children and young people in education settings (e.g., respectful relationships training). Aligned with this definition of a primary prevention intervention, our search strategy was broad in scope and included interventions that were explicitly described as primary prevention of violence against women, as well as violence prevention programming that addressed risk factors for violence against people with disability given their potential relevance to preventing gendered disability violence.

### Search Strategy

We identified relevant studies in the international peer-viewed literature by systematically searching the bibliographic databases of Medline, CINAHL, Embase, and PsychInfo. Search terms, tailored to requirements of the different databases, were developed in collaboration with a trained research librarian and comprised the following Medical Subject Headings terms, keyword combinations, and Boolean operators: (disability OR disabled OR disabled persons OR impaired OR impairment) AND (violence OR exposure to violence OR family violence OR domestic Violence OR intimate partner violence AND (primary prevention OR prevention OR secondary prevention OR early intervention OR program evaluation OR strategies OR best practices OR treatment OR therapy OR program OR management). The search terms used were deliberately broad to increase the sensitivity of the search and identify all possible studies. We also reviewed the reference lists of all included studies and relevant reviews to identify any further publications not uncovered in the database searches. Searches were undertaken in May and June 2020 and updated in December 2020.

### Eligibility Criteria and Study Selection

Included studies had to be an evaluation or analysis of effectiveness of a primary prevention intervention. Interventions could be designed to prevent any form/s of violence (e.g., physical violence, sexual violence) by any perpetrator (e.g., intimate partner, other family member, carer, stranger) addressing any type/s of disability or impairment (e.g., physical, sensory, speech, cognitive/intellectual, psychological/psychosocial). Studies reporting quantitative outcomes from evaluated interventions based on any study design were included. Inclusion was restricted to studies in which primary prevention was the main focus of the intervention. In some programmatic content areas, for example, sexuality and relationship education, studies were considered eligible if the primary focus of the intervention was prevention (e.g., knowledge, safety, privacy, consent) rather than biological content only (e.g., anatomy, development, puberty, reproduction). All papers included were written in English and published on or after January 1, 2010.

Interventions that had not been subject to an evaluation or reported on process or implementation outcomes only (i.e., fidelity) were excluded. Studies were also excluded if they were theoretical, opinion pieces, commentaries, books, book chapters, published conference abstracts, systematic reviews and meta-analyses, or protocol papers. Studies in which the focus of the intervention was on responding to violence after it had occurred were excluded. Studies of interventions that combined primary prevention and early intervention were considered eligible if the stated aim was to prevent violence from happening rather than responding after violence had occurred. Studies were considered ineligible if they focused exclusively on the prevention of violence against children (child abuse and maltreatment) or those that were designed to prevent violence against men only.

One author (LK) carried out the initial screening of study titles and abstracts. Two authors (LK and GS) then independently reviewed the full text version of studies that met the inclusion criteria. Any conflict about a paper’s relevance for inclusion was resolved by discussion and consensus between the two review authors.

### Data Extraction and Analysis

Data from included studies were extracted according to prespecified criteria and included country of origin; description of the intervention (including type, setting, content mode of delivery); study design; participant characteristics and sample size; methods and measures; and summary of outcomes. Data were extracted by one author (LK) and reviewed by a second author (GS) with any discrepancies resolved by discussion. Information extracted was synthesized in a narrative review.

### Assessment of Quality

The Quality Assessment Tool for Quantitative Studies (QATQS) developed by the Effective Public Health Practice Project was used to appraise the quality of evidence in each included study (QATQS-EPHPP, 2013). This tool was selected because it is applicable to evaluations using a range of study designs and has previously been applied to studies assessing the effectiveness of violence prevention interventions (Mikton et al., 2014; Stobbe et al., 2021). Studies were assessed according to the QATQS eight domains of bias including selection bias, study design, confounding, blinding, data collection method, withdrawal and drop out, intervention integrity, and analysis appropriate to the research question, with each domain graded as strong, moderate, or weak in accordance with the QATQS detailed tool guide and dictionary. As per the QATQS, studies were then assigned a global rating of “weak” if two or more domains of bias were rated as weak; “moderate” if one domain was rated as weak; and “strong” if no study domains received a weak rating. Assessment of quality was undertaken independently by two authors (GS and LK), with one discrepant rating resolved by discussion.

## Results

**Figure 1.**
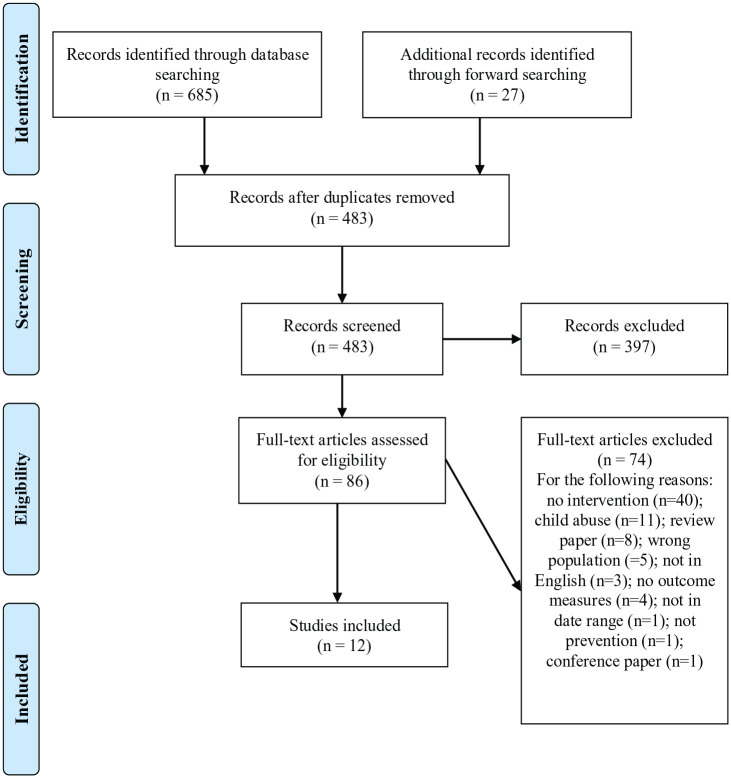
PRISMA flow diagram.

The search strategy identified 483 studies of potential interest (after the removal of duplicates). After screening the title and abstracts, the full text of 86 studies was assessed. A total of 12 studies were retained (Figure 1).

### Characteristics of Included Studies

The characteristics of the selected studies are shown in Table 1.

**Table 1.** Characteristics of Included Studies.

Author (year)/Country	Intervention	Design	Participants	Main Outcomes	Key Findings	QATQS Rating
Bowman et al. (2010)/USA	Sexual abuse prevention workshop consisting of 4 hr of face-to-face training	Pre- and post-test design	*N* = 124 developmental disability service providers from West Virginia	Knowledge and attitudes included the following surveys: Sexual Abuse Attitudes and Knowledge Questionnaire and the Global Perceptions Scale Data were collected via surveys with participants before and immediately after training	Results showed small improvements in knowledge and attitudes about sexual abuse and the sexuality of persons with developmental disability; however, general attitudes about individuals with developmental disability did not change from pre- to post-training	Weak
Devries et al. (2018)/Uganda	*Good School Toolkit*—a school-based intervention to reduce physical violence from school staff to primary school students and peer-to-peer violence implemented over 18 months	Cluster RCT	*N* = 42 primary schools in Luwero District, Uganda, were randomly assigned to receive the Good School Toolkit for 18 months, or to a wait-list control group. *N* = 2,956 students comprising *n* = 644 with functional difficulty in one domain; *n* = 220 with disability	Past week physical violence with data collected from school staff and school students in primary years 5, 6, and 7 (aged 11–14 years) at baseline and post-intervention (i.e., at conclusion of 18-month program) using the International Society for the Prevention of Child Abuse and Neglect Child Abuse Screening Tool-Child Institutional	Results showed reductions across a range of different forms of violence from staff and peers toward students, including among students who reported no functional difficulties, those who reported some difficulty in one domain, and those who reported a disability	Moderate
Dryden et al. (2014)/USA	*IMPACT: Ability*—a 10 × 90-min weekly classroom-based program for safety and self-advocacy training for people with cognitive and/or physical disabilities	Nonrandomized controlled trial	*N* = 57 students across five special education high schools in Boston comprising an intervention (*n* = 21) and wait-list control group (*n* = 36). Students were 58% males and 82% non-white, with an average age of 17 years. All students were reported as having intellectual disabilities	Safety and self-advocacy knowledge, confidence in self-protection, self-determination, feelings of safety, self-advocacy, and general self-efficacy. Data were collected by surveys in the week prior to and after program completion	Results showed significantly greater improvement in key outcomes, including safety and self-advocacy knowledge, confidence, and behavior for intervention students compared to the wait-list group. Results also showed evidence of improvements in students’ sense of safety and general self-efficacy	Weak
Dryden et al. (2017)/USA	*IMPACT: Ability* (see Dryden et al., 2014)	Pre- and post-test design (follow-up)	*N* = 47 of the 57 students who took part in the program (as per Dryden et al., 2014)	Safety and self-advocacy knowledge, confidence in self-protecting, self-determination behaviors, feelings of safety, self-advocacy, and general self-efficacy. Data were collected via survey at 12 months post completion of the course	Results showed significant post-training improvements in participants’ safety and self-advocacy knowledge and confidence were maintained 1-year later	Weak
Ejaz et al. (2017)/USA	Online training comprising three modules: (1) knowledge of different types of abuse, poly-victimization, prevalence and common characteristics of victims and perpetrators; (2) screening for abuse, communication, identifying those at risk and (3) reporting protocols	Pre- and post-test design	*N* = 273 service providers completed at least one module; *n* = 212 completed all three. By profession, most participants were social workers, counselors, nurses, or nurse practitioners	Changes in knowledge in how to identify and report abuse, exploitation, and neglect. Data were collected via surveys with participants before and immediately after training	Results showed improvements in knowledge from pre- to post-training on abuse and reporting abuse, but not for communication principles and screening for abuse	Weak
Fisher et al. (2013)/USA	Multicomponent intervention that consisted of instructions, modeling, rehearsal, and feedback conducted in the classroom. In situ training was conducted in three different community settings One follow-up booster session for a selection of participants	Pre- and post-test design	*N* = 5 young adults with intellectual disabilities	Participant behavior in relation to appropriate responses to lures observed in the classroom, in situ and during the booster session. Data were collected through in-session and in situ assessments of participants, with outcomes observed by the simulated stranger or trainer	Results showed a training effect whereby participants did not walk from strangers who tried to lure them prior to training, but demonstrated appropriate responses during classroom and in situ training	Weak
Hickson et al. (2015)/USA	The *Effective Strategy-Based Curriculum for Abuse Prevention and Empowerment* (ESCAPE-DD)—a curriculum comprising two units: developing an understanding of abuse concepts and the acquisition of a four-step, reasoning-based, effective decision-making strategy	RCT	*N* = 58 women and men with mild and moderate intellectual and developmental disability recruited from seven adult day program sites in New York City. The average age of the sample was 39 years, and the mean IQ was 57. Most participants lived at home with their families	Decision-making in relation to relationships and violence using the Decision-making Scale. Data were collected via structured individualized interviews prior to and approximately 1 week after the completion of the intervention	Results showed that participants in the intervention group made significantly greater gains on measures of overall effective decision-making and safe-now effective decision-making relative to participants in the control group	Moderate
Hughes et al. (2010)/USA	*A Safety Awareness Program for Women with Disabilities* (ASAP for Women)—8 × 2.5-hr weekly classes with didactic and interactive components, including weekly action planning with group feedback and problem solving	Pre- and post-test design	*N* = 7 women with diverse disabilities including physical, cognitive, speech, or unspecified. The sample was reported as comprising middle-age, unmarried, white women	Abuse awareness, safety self-efficacy, safety skills, social support/isolation, and safety promoting behaviors. Data were collected via class evaluations administered immediately prior to and at the conclusion of the last session	Results showed significant increases from baseline to post-intervention on measures of self-efficacy and safety skills. Although not statistically significant, improvements were also found in safety promoting behavior	Weak
Robinson-Whelen et al. (2010)/USA	*Safer and Stronger Program* (﻿SSP)—computer-based assessment and intimate partner violence self screening tool	RCT	*N* = 305 women with disability who completed the SSP at T1 (*n* = 172) and the control group who completed the SSP at T2 (*n* = 133). Participants were reported as having diverse disabilities (including mental and physical health conditions/disability, vision, hearing and speech impairment, cognitive/learning disability), with most reporting more than one disability	Abuse awareness, safety self-efficacy, safety promoting behaviors. Data were collected via questionnaire at the time of the online training and again 3 months later	Results demonstrated the intervention group had greater abuse awareness than the control group at T2, and abuse awareness increased from T1 to T2 among women in the intervention group, particularly among women who had experienced little or no abuse in the past year . Both abuse awareness and safety self-efficacy were significantly related to safety behaviors	Moderate
Robinson-Whelen et al. (2014)/USA	*ASAP for Women* (see Hughes et al., 2010)	RCT	*N* = 213 women with disability recruited through 10 centers for independent living, CIL) comprising *n* = 109 in the intervention group and *n* = 104 assigned to usual care. Participants were reported as having diverse disabilities, including mental and physical health conditions/disability, vision impairment, cognitive/learning disability	Abuse awareness, abuse and safety knowledge and skills, safety self-efficacy, social network/support, and safety promoting behaviors. Data were collected via questionnaire at three time points: at baseline (prior to the intervention), at 2 months (immediately after the intervention), and at 6 months post-intervention	Results showed that in comparison to the control group, women in the intervention arm improved on almost all measures of protective factors including abuse awareness, abuse and safety knowledge, safety skills, safety self-efficacy, social support, and safety promoting behaviors. Outcomes were maintained 6 months after completion of the program	Moderate
Hughes et al. (2020)/USA	*The Safety Class*—8× weekly face-to-face classes with didactic and interactive components, including communication skills, respectful relationships, safety planning, help seeking, and disability rights	RCT	*N* = 170 adult women and men with intellectual disability recruited through 12 CIL comprising *n* = 95 in the intervention and *n* = 89 assigned to the control group. Participants had a mean age of 33 years (range 18–67). The majority were white; a quarter were Hispanic	Abuse knowledge and awareness; knowledge of healthy relationships; warning signs; safety, communication, and safety planning skills; safety self-efficacy. Data were collected via interviewer administered surveys at pre-, immediately post, and 3 months post-intervention	Results showed that in comparison to the control group, participants in the intervention arm improved their knowledge about safety and abuse and safety and communications skills. This was maintained at 3 months post program. There was no commensurate significant shift for knowledge of healthy relationships, warning signs of abuse, safety planning skills, safety self-efficacy	Weak
Ward et al. (2013)/USA	*The Friendships and Dating Program (FDP)*—20 sessions × 10 weeks designed to prevent violence in dating and partnered relationships and to teach social skills needed to develop healthy relationships	Pre- and post-test design	*N* = 31 adult women and men with intellectual and developmental disability were recruited by five community agencies in Alaska.	Social network size and experience of violence with data collected via face-to-face interviews with facilitators at baseline, after the completion of the program, and 10 weeks following the end of the program	The results showed the size of the participants’ social networks increased and the number of incidents of interpersonal violence was reduced for participants who completed the FDP. Outcomes were maintained 10 weeks later	Weak

*Note*. RCT = randomized controlled trials; QATQS = Quality Assessment Tool for Quantitative Studies.

### Country of Origin

All but one study was conducted in a high-income country. Ten of the twelve studies were conducted in the USA and one in the Republic of Korea. One study was conducted in Uganda which is categorized as a lower middle-income country.

### Participant Characteristics

Seven studies assessed the effectiveness of primary prevention of violence interventions with adults with disability (Fisher et al., 2013; Hickson et al., 2015; Hughes et al., 2010, 2020; Robinson-Whelen et al., 2010, 2014; Ward et al., 2013); three of which included only women with disability as participants (Hughes et al., 2010; Robinson-Whelen et al., 2010, 2014). In a further three studies, the interventions were with school-aged children (Devries et al., 2018; Dryden et al., 2014, 2017; Ejaz et al., 2017). Two studies evaluated interventions targeting disability service providers/support workers and one study reported on an intervention delivered to students and staff in a school setting (Bowman et al., 2010; Ejaz et al., 2017). Among the nine studies that included people with disability as participants, three interventions were described as being specifically for people with intellectual or developmental disability (Fisher et al., 2013; Hickson et al., 2015; Hughes et al., 2020). The remaining studies either described participants as having diverse disabilities usually comprising mental and/or physical health conditions, hearing, speech, and visual impairment, and/or cognitive/learning disability.

### Study Design

Studies employed a range of evaluation designs. The most widespread was a nonrandomized, noncontrolled pre- and post-test design in which outcomes were measured before and after the intervention. One study used a nonrandomized wait-list controlled trial design (Dryden et al., 2014, 2017), four were randomized controlled trials (RCT; Hickson et al., 2015; Hughes et al., 2020; Robinson-Whelen et al., 2010, 2014), and one employed a cluster RCT design (Devries et al., 2018). A cluster RCT differs from a standard RCT in that the unit of randomization is at the group rather than individual level, for example, a school, workplace, or health service.

Studies used quantitative methods to collect data including surveys, structured interviews, or structured observations through role-play or in situ scenarios. Some were mixed methods using a combination of surveys, interviews, focus groups, and observational data. Measures of effectiveness largely focused on outcomes related to risk and protective factors including awareness, knowledge, attitudes, skills in safety, and self-protective behaviors (i.e., understanding and knowing how to enact learned strategies to safeguard against violence) and confidence to identify and report violence. Only two studies measured self-reported experiences of violence (Devries et al., 2018; Ward et al., 2013).

Sample size ranged from 5 to 305. The cluster RCT conducted by Devries et al. (2018) included a sample of 2,956 students, but the unit of analysis was at the level of the school (*N* = 42). Overall, the included studies had relatively small samples with 6 of the 13 studies reporting less than 50 participants.

### Assessment of Quality

Eight of the twelve studies were assessed as having a weak global rating on the QATQS because they were rated as weak on two or more risk of bias domains (Bowman et al., 2010; Dryden et al., 2014, 2017; Ejaz et al., 2017; Fisher et al., 2013; Hughes et al., 2010, 2020; Ward et al., 2013). Most of the studies that received a weak global rating were assessed as weak on at least four of the eight domains of the QATQS, largely due to study design. For example, studies that employed nonrandomized, noncontrolled pre- and post-test designs were rated as weak on the risk of bias domains pertaining to the selection of study participants, design, confounding and intervention integrity (Bowman et al., 2010; Ejaz et al., 2017; Fisher et al., 2013; Hughes et al., 2010, 2020; Ward et al., 2013). In these studies, the validity of the data collection methods was either not described or could not be determined. Dryden et al. (2014, 2017), who used a pretest design with wait-list control, received a moderate rating for design, a strong rating on confounding but weak for the remaining domains. Four studies were assessed as having a moderate global rating with one domain of bias graded as weak (Devries et al., 2018; Hickson et al., 2015; Robinson-Whelen et al., 2010, 2014). In all these studies, group allocation (control or intervention) was not concealed from outcome assessments or participants, or blinding was not adequately described, resulting in a weak rating on this domain of bias. No studies received a strong global rating.

### Characteristics of Interventions

The 12 included studies reported on 10 different primary prevention interventions or programs (i.e., two studies reported on *IMPACT: Ability* and two studies reported on *ASAP for Women*). The 10 programs varied in content, scope, duration, and mode of delivery. Below we describe intervention characteristics and evidence of quality and effectiveness according to the specified target population.

#### Women With Disability

Three studies reported on two primary prevention interventions for women with disability—the *Safer Stronger Program* (SSP) and *A Safety Awareness Program for Women* (ASAP for Women) (Hughes et al., 2010; Robinson-Whelen et al., 2010, 2014). Both programs were described as addressing the high rates of interpersonal violence experienced by women with disability, and articulated how the intervention was designed to bring about change including by drawing on the theoretical frameworks of feminist disability theory. Both interventions were information- and skills-based programs that focused on building the capacity of women with disability to understand and recognize violence and abuse and enact safety and self-protective skills. One intervention (ASAP for Women) was peer led. Two of these three studies reported on the outcomes of RCTs and were rated as moderate on the QATQS (Robinson-Whelen et al., 2010, 2014).

Robinson-Whelan et al. (2010) evaluated the SSP, which is a multicomponent internet-based abuse awareness and safety program. In this study, a total of 305 women with diverse impairment types were randomly allocated to the intervention group who completed the SSP (*n* = 172) twice with a 3-month interval, or to the control group (*n* = 133), who completed the SSP at the 3-month time point only. Those in the intervention group reported significantly greater knowledge and abuse awareness at 3 months, in comparison to participants in the control group. There was no commensurate shift in participants’ capacity to enact learned strategies to safeguard against violence (called self-protective or safety behaviors).

The remaining primary prevention intervention for women with disability was ASAP for Women, which is a multi-session, peer-led program delivered in a residential group home setting. An earlier pilot study had demonstrated acceptability of this program for building safety behaviors (Hughes et al., 2010), and later Robinson-Whelan et al. (2014) evaluated the program using an RCT. Results showed that in comparison to the usual care comparison group (*n* = 104), women who participated in the 8-week program (*n* = 109) reported significant improvements in safety promoting behaviors, skills, knowledge, and confidence to enact strategies to stay safe and avoid abusive situations, as well as improved social networks.

#### Children, Young People, and Adults With Disability

In six studies, mixed gendered groups of children, young people, or adults with disability were the target population (Dryden et al., 2014, 2017; Fisher et al., 2013; Hickson et al., 2015; Hughes et al., 2020; Ward et al., 2013). In these studies, the evaluated interventions were primarily information, behavior, and/or skills based, delivered in specialist education, residential or other disability service settings. These interventions were focused on building the capacity of children, young people, and adults with disability to understand and recognize violence and enact safety skills and self-advocacy. Most programs were group based and were delivered as multi-session interventions.

While several of these six studies reported improvements from pre- to post-intervention on participant self-reported or observed outcomes, five received a global rating of weak on the QATQS with a high risk of bias identified across several study domains including selection of participants, confounding, blinding, and intervention integrity (Dryden et al., 2014, 2017; Fisher et al., 2013; Hughes et al., 2010; Ward et al., 2013). Additionally, many of these studies included only small numbers of participants, did not include a comparison group, and/or did not measure outcomes beyond the immediate completion of the program.

Only one study among this group used exposure to violence as an outcome. This study was an evaluation of the *Friendships and Dating Program*—a skills-based intervention for men and women with intellectual and developmental disability to build and develop healthy intimate and non-intimate relationships (Ward et al., 2013). The program consisted of 20 sessions delivered over a 10-week period. Seventeen men and fourteen women (total *n* = 31) completed facilitator-assisted semi-structured interviews at baseline, post program completion, and at 10 weeks follow-up. Results showed an increase in participants’ social network size and a significant reduction in self-reported incidents of interpersonal violence from baseline to immediate post-course, with a reduction in interpersonal violence maintained at 10 weeks follow-up. This study, however, was assessed as “weak” with five of the eight individual domains of bias on the QATQS rated as weak.

#### People Who Work With and/or Support People With Disability

Two studies focused on people who work with and/or support people with disability (Bowman et al., 2010; Ejaz et al., 2017). Bowman et al. (2010) described the use of a 4-hr sexual abuse prevention workshop for disability service providers. While the study reported small improvements in knowledge and attitudes about sexuality and sexual abuse of adults with development disability from pre- to post-training, participants’ general (negative) attitudes toward people with disability did not change. The study by Ejaz et al. (2017) evaluated an online training program for disability support managers which addressed identifying and reporting abuse. Results showed that knowledge about abuse and how to report it improved from pre- to post-training. Both interventions were delivered as a one-off session and data were collected before and immediately after training with no follow-up. The studies were assessed as weak on the QATQS with a high risk of bias identified across at least five domains of bias.

#### Whole of Community

The remaining study of the 12 included in this review used a cluster RCT to evaluate the effectiveness of a whole of community physical violence prevention intervention delivered in a school setting (Devries et al., 2018). The *Good Schools Toolkit* is a school-based behavioral intervention delivered in primary schools over an 18-month period. This study by Devries and colleagues was conducted in one district of Uganda with the aim of reducing physical violence in the student and staff population and included a focus on reducing violence toward children with disability. Results showed the intervention reduced a range of different forms of staff to student and peer violence with no difference between children with or without disability. This study received a moderate global rating with only one domain of bias (blinding as part of a controlled trial) graded as weak.

## Discussion

The focus of this systematic review was to identify and synthesize research on evaluated interventions addressing the primary prevention of violence against women with disability and explore evidence about their quality and effectiveness. The search strategy was deliberately broad to capture the range of possible approaches to primary prevention from those targeting individual behavior change to those addressing socio-structural and cultural norms. From a total of 483 studies, we identified 12 peer-reviewed publications in the scholarly literature for inclusion. Our narrative review of these studies allowed us to consider the current state of knowledge on the primary prevention of violence against women with disability, including the type and content of interventions, evidence of effectiveness, the extent to which they respond to the known drivers of violence, and study quality.

Most studies were assessed as being at high risk of bias with 8 of the 12 included studies classified as weak on the global rating of the QATQS. However, unlike the most recent systematic review by Mikton et al. (2014), in which all the included studies received a weak rating, we found a selection of higher quality studies employing more robust methodological designs including RCTs. Over the 10-year time frame of this review (2010–2020) several interventions first reported on process or feasibility indicators (not meeting the inclusion criteria for this review), followed by a further trial of outcome indicators such as *ASAP for Women* (Hughes et al., 2010; Robinson-Whelen et al., 2014). This general shift over time toward more rigorous evaluation methodology is a promising sign in terms of both program development and for the strength of the scholarly literature overall. Progress is dependent on research initiatives that build on the existing evidence base and move toward identifying what works for whom and under what circumstances.

Overall, however, this review illustrates that the evidence base on the primary prevention of violence against women with disability remains substantially underdeveloped with key knowledge gaps. Assessing the extent to which studies can be deemed effective in preventing violence from occurring is difficult to establish, but it is made more difficult by studies that conflate primary and secondary, or even tertiary prevention. In addition, most studies measured outcomes immediately after the program’s completion only or had short follow-up periods. Both interventions targeting disability service providers were delivered as a brief, one-off session (Bowman et al., 2010; Ejaz et al., 2017) despite evidence that such an approach is unlikely to effect change (Jewkes et al., 2021). Only one study explicitly described that the intervention or program being evaluated was developed and/or informed by women with disability (Robinson-Whelen et al., 2010). It appears that most interventions in the peer-reviewed literature fail to draw on the existing knowledge base about the underlying drivers of violence against women with disability (Our Watch & Women with Disabilities Victoria, 2022) or the characteristics of effective primary prevention practice (Jewkes et al., 2021). Most studies did not articulate a theoretical framework underpinning the intervention design and implementation, nor did they describe the mechanisms by which the intervention was intended to bring about change.

Primary prevention entails looking “upstream” to address factors that lead to the perpetration of violence. Yet, the most well-developed area of research is evaluations of interventions that attend to a narrow set of risk factors for violence located at the individual level of the socio-ecological model. That is, interventions that focus on raising awareness about abuse, recognizing violent behaviors, and promoting the use of protective and self-advocacy skills for people with disability; many specifically designed for women. While these studies showed evidence of effectiveness for achieving intended outcomes, the interventions themselves are problematic because they focus the responsibility for preventing violence on women with disability. There is an urgent need to shift the obligation from women with disability to protect themselves from violence and place the responsibility for women’s safety back onto services and systems.

While raising awareness and increasing knowledge about violence and promoting safety behaviors for women should not be dismissed as unimportant, prevention strategies that target individual risk only are unlikely to result in significant or sustained social change without a commensurate and coordinated focus across multiple levels of influence in the socio-ecological model. We acknowledge that interventions geared toward addressing community and institutional practices and systematic risk factors for violence are much more difficult to develop, implement, and evaluate, and require long-term investment. Despite the challenges they pose, interventions addressing the primary prevention of violence must move beyond individualized approaches and focus on the policies, practices, and systems that enable and normalize violence against women with disability. This may include, for example, targeting community attitudes and negative stereotypes about women (and all people) with disability; attending to the institutionalized power asymmetries between women with disability and support and social services; and addressing laws and policies that exclude women with disability from decision-making spaces.

The studies identified in this review lacked a focus on diversity and social identity beyond disability. The language and concepts used in the studies to describe the “problem” of violence against women with disability mostly failed to consider the underlying drivers of violence that sit at the intersection of ableism, sexism, and other forms of discrimination and exclusion based on factors such as class, race, and sexuality. Consideration of intersectionality or the extent to which overlapping systems of inequality and discrimination might impact on approaches to preventing violence against women with disability was largely absent from the literature.

The available literature, for example, ignores the experiences of trans and gender diverse people, both in relation to violence and intervention effectiveness. Despite evidence that trans and gender diverse people experience high rates of violence and abuse and face specific forms of discrimination across multiple social contexts (Amos et al., 2023), research on violence against women remains largely reliant on a cisgender paradigm.

It is also important to note that women with disability are not homogenous group. Experiences of violence differ according to a range of socio-demographic characteristics including impairment type and age (Mailhot Amborski et al., 2022). Most studies identified in this review, however, did not acknowledge this potential diversity or consider the impact on intervention effectiveness. There are a range of reasons why we cannot draw meaningful conclusions about the moderating role of impairment type or other characteristics including the variability of recruitment into studies, as well as the basis for identifying participants as having a disability. The critical findings of this review are summarized in Table 2.

**Table 2.** Summary of Critical Findings.

Research shows that women with disability experience violence and abuse at significantly higher rates than their nondisabled peers, but we know much less about “what works” to prevent it from happening in the first place (i.e., primary prevention).
There was a relatively small pool of 12 studies available in the peer-reviewed academic literature to assess the evidence: Eight of them receiving a “weak” global rating for quality due to a high risk of bias.
The evidence base on the primary prevention of violence against women with disability remains substantially underdeveloped.
Most interventions targeted people with disability and aimed to raise awareness about violence, improve participants’ knowledge and skills to recognize violence, and enact safety and self-advocacy behaviors.
Primary prevention strategies that target individual-level risk in isolation are unlikely to result in significant or sustained social change without a commensurate and coordinated focus across multiple levels of influence including at the relationship, community, and societal levels.

### Limitations

Primary prevention of violence against women is a distinct approach that focuses on addressing the underlying factors that lead to and enable violence to occur. As such primary prevention action is both broader in scope and conceptually different from interventions that respond to existing violence or violence after it has occurred. Studies included in this review, therefore, reflected this distinct yet broad approach. This review had several limitations. First, we were not able to include studies published in a language other than English. Second, all but one study reported on interventions conducted in high-income countries; most were from the USA. Third, in some studies it was difficult to identify the focus of the intervention. This was particularly the case when studies evaluated interventions that combined primary and secondary prevention (e.g., Ejaz et al., 2017). Fourth, it may be the case that a range of relevant literature was not included in the review because interventions at a structural level, for example, increasing women’s access to housing or financial resources may not always be described in the literature as primary prevention, and therefore was not picked up by the search terms used. Fifth, we restricted our search to peer-reviewed papers in the scientific literature. This meant that community-generated research reports, sometimes referred to as “gray” literature, were not included. Given efforts to design and implement interventions to prevent violence against women with disability may occur through government or community organizations, there are likely to be additional and relevant program evaluations that sit in within the “gray” literature.

## Conclusion

The need for robust evidence on what works for whom and in what context to prevent violence against women with disability is urgent and long overdue. Our review contributes by systematically reviewing the available evidence base. However, only 12 studies that reported on the effectiveness of primary prevention interventions were found. Most interventions targeted people with disability and aimed to improve participants’ knowledge and skills to recognize violence and enact safety and self-advocacy behaviors. Most studies reported outcomes using pre- and post-test methodologies and received a weak quality assessment rating on the QATQS.

For women with disability the drivers of some of the violence they experience are likely to be similar to those experienced by women without disability, but with different and additional drivers of violence at the intersection of gender- and disability-based discrimination (Dowse et al., 2016). This review of the international peer-reviewed literature on the effectiveness of interventions to prevent violence against women with disability, however, suggests a “disconnect” between emergent perspectives on what drives violence, intervention, and program development and empirical insights derived from evaluation. As summarized in Table 3, this has several implications for policy and practice.

**Table 3.** Implications for Policy and Practice.

There is a body of evidence showing the importance of attending to the intersection of gender and disability inequality to prevent violence against women with disability.
Policies and practices in primary prevention must prioritize evidence-informed approaches by targeting ableism and sexism, and their intersection, across multiple levels of the socio-ecology—individual, relationship, community, and society.
It is important that action to prevent violence does not frame the issue as one that women with disability are responsible for “fixing.”
Primary prevention interventions must prioritize the authentic inclusion of people with disability in design, development, implementation, and evaluation. This is currently a significant gap.

Understandings of how and why the problem of violence against women with disability arises have been slower to develop than in nondisabled populations. However, there is now an emerging body of evidence showing the importance of attending to the intersection of gender and disability inequality for effective intervention. Policies and practices in primary prevention, therefore, must prioritize the social context that enables violence against women with disability to happen. This means targeting ableism and sexism and the way these interact and manifest across multiple levels of the socio-ecology—individual, relationship, community, and society. Interventions targeting issues of empowerment, community engagement, social inclusion, decision-making and the safe and respectful provision of disability, support, and other services show promise in terms of their primary prevention potential (Cramer, 2014) but have not been subject to rigorous evaluation designs with behavioral outcome measures. Yet, the current evidence base, as synthesized in this review, remains focused almost exclusively on programming and practices that attend to drivers or risk factors for violence at the individual level only. While raising awareness about violence and teaching skills so that women can recognize, report, and protect themselves against violence represents one possible avenue for prevention, it is important that action to prevent violence does not frame the issue as one that women with disability are responsible for “fixing.” Rather strategies to address the primary prevention of violence against women with disability should focus on social and cultural norms, structural and systematic discrimination, and discriminatory and exclusionary practices in organizations, families, and relationships.
